# Potential In Vitro Inhibition of Selected Plant Extracts against SARS-CoV-2 Chymotripsin-Like Protease (3CL^Pro^) Activity

**DOI:** 10.3390/foods10071503

**Published:** 2021-06-29

**Authors:** Carla Guijarro-Real, Mariola Plazas, Adrián Rodríguez-Burruezo, Jaime Prohens, Ana Fita

**Affiliations:** Instituto de Conservación y Mejora de la Agrodiversidad Valenciana, Universitat Politècnica de València, 46022 Valencia, Spain; carguire@etsia.upv.es (C.G.-R.); adrodbur@upvnet.upv.es (A.R.-B.); jprohens@btc.upv.es (J.P.); anfifer@btc.upv.es (A.F.)

**Keywords:** allyl isothiocyanate, COVID-19, curcumin, FRET assay, mustard seeds, natural compounds, plant extracts, SARS-CoV-2 3CL^Pro^, turmeric, wall rocket

## Abstract

Antiviral treatments inhibiting Severe acute respiratory syndrome coronavirus 2 (SARS-CoV-2) replication may represent a strategy complementary to vaccination to fight the ongoing Coronavirus disease 19 (COVID-19) pandemic. Molecules or extracts inhibiting the SARS-CoV-2 chymotripsin-like protease (3CL^Pro^) could contribute to reducing or suppressing SARS-CoV-2 replication. Using a targeted approach, we identified 17 plant products that are included in current and traditional cuisines as promising inhibitors of SARS-CoV-2 3CL^Pro^ activity. Methanolic extracts were evaluated in vitro for inhibition of SARS-CoV-2 3CL^Pro^ activity using a quenched fluorescence resonance energy transfer (FRET) assay. Extracts from turmeric (*Curcuma longa*) rhizomes, mustard (*Brassica nigra*) seeds, and wall rocket *(Diplotaxis erucoides* subsp. *erucoides*) at 500 µg mL^−1^ displayed significant inhibition of the 3CL^Pro^ activity, resulting in residual protease activities of 0.0%, 9.4%, and 14.9%, respectively. Using different extract concentrations, an IC_50_ value of 15.74 µg mL^−1^ was calculated for turmeric extract. Commercial curcumin inhibited the 3CL^Pro^ activity, but did not fully account for the inhibitory effect of turmeric rhizomes extracts, suggesting that other components of the turmeric extract must also play a main role in inhibiting the 3CL^Pro^ activity. Sinigrin, a major glucosinolate present in mustard seeds and wall rocket, did not have relevant 3CL^Pro^ inhibitory activity; however, its hydrolysis product allyl isothiocyanate had an IC_50_ value of 41.43 µg mL^−1^. The current study identifies plant extracts and molecules that can be of interest in the search for treatments against COVID-19, acting as a basis for future chemical, in vivo, and clinical trials.

## 1. Introduction

Severe acute respiratory syndrome coronavirus 2 (SARS-CoV-2) is a predominantly airborne transmitted coronavirus first identified in December 2019, which is the causal agent of Coronavirus disease 19 (COVID-19) [1]. Rapid spread of SARS-CoV-2 has caused a severe pandemic, resulting so far in a death toll of almost 3 million lives worldwide [2] and a disruption of many aspects of human daily life. SARS-CoV-2 is an enveloped, simple-stranded, positive-sense RNA virus with a length of 29.9 kb and a diameter of about 65–125 nm [3]. Since its identification, the genome of multiple strains of SARS-CoV-2 has been sequenced [4,5,6] and its life cycle has been thoroughly studied [3].

Many vaccine projects have been developed and are underway to fight COVID-19 [7]. Several of the first-generation vaccines already in use in vaccination campaigns have shown high levels of efficacy, albeit the level of protection is not complete and seems to be strain-dependent. In this way, the recent appearance of new highly infectious SARS-CoV-2 strains might reduce the efficacy of first-generation vaccines [8]. Moreover, vaccines are still in short supply and, in many countries, vaccination against SARS-CoV-2 is neither compulsory nor indicated for some specific groups, such as children, or people with certain medical conditions [9,10]. Therefore, the development of therapies complementary to vaccination is desirable for a comprehensive fight against COVID-19.

The development of drugs for the treatment of COVID-19 patients so far has not resulted in highly efficient or curative drug therapies. Despite the initial consideration of some pre-existent drugs as potential antiviral against SARS-CoV-2, no proven effective treatments have been found so far [11]. Among the proteins encoded by the SARS-CoV-2 RNA, the chymotrypsin-like protease (3CL^Pro^), also known as main protease (M^Pro^), is required for the proteolytic processing of the viral polyproteins during the maturation step, thus becoming an essential tool for the successful replication of the virus [12]. In this sense, the fundamental role of the 3CL^Pro^ in viral replication makes this enzyme an attractive target for the development of drugs inhibiting the virus replication. A similar research strategy was followed in the past against the phylogenetically related SARS-CoV [13], which caused the SARS-CoV 2002–2004 outbreak, and led to the screening of a large variety of molecules and natural products for their inhibition of the SARS-CoV 3CL^Pro^ activity [14]. In addition to single molecules, some plant extracts also inhibited the SARS-CoV 3CL^Pro^ activity in vitro [15,16].

Some natural compounds with potential against SARS-CoV-2 have been identified based on the work with SARS-CoV and related viruses and through in silico molecular docking studies [17,18,19,20,21]. The recent work of Zhou and Huang [22] reviews the current findings regarding natural compounds that might be considered for their potential antiviral activity against SARS-CoV-2, mostly based on previous experience with other coronaviruses. Some compounds found in edible and/or medicinal plant products were identified in these studies as potential natural compounds targeting the SARS-CoV-2 3CL^Pro^, including glucosinolates, flavonoids, and other phenolic compounds. Phenolic compounds have been extensively studied for their antioxidant capacity acting as free radical scavengers tested in vitro and using in vivo models [23]. Thus, diets rich in fruits and vegetables containing high levels of these compounds are associated with a reduction in the risk of chronic inflammatory processes, thus lowering the risk of developing some degenerative and cardiovascular diseases and several types of cancer, and increasing the overall health status [24,25,26]. In addition, glucosinolates and their hydrolytic products are also bioactive molecules related to the maintenance of a good health status. The most studied derivative, sulforaphane, proved to induce detoxification phase II enzymes [27] and has been studied as an anticarcinogen likely able to be effective in all stages of cancer [28], to reduce the risk of developing type 2 diabetes [29], as well as to ameliorate several neurodegenerative diseases [30]. Other glucosinolates are promising as natural compounds by exerting antimicrobial activities [31,32].

However, the evaluation of the antiviral capacity of these groups of natural compounds is more limited. Based on this consideration, the current work aimed at the targeted testing for inhibitory capacity against the 3CL^Pro^ activity of plant extracts selected for being rich in one or more of the aforementioned chemical families. The materials selected constitute common food products in many cultures and are of easy access or, alternatively, grow profusely in many regions and are included in traditional cuisines, as is the case of wall rocket in the Mediterranean region. A cell-free cleavage assay using a fluorogenic substrate was used, as this technique has been used successfully to measure the protease activity of the related SARS-CoV 3CL^Pro^ [33,34,35]. Testing plant extracts can be considered a first approach in the search for natural compounds with antiviral activity, or even represent a basis for the development of prophylactic or therapeutic plant extracts against COVID-19. In addition, given the proven safety for human consumption of the plants from which extracts are obtained, their potential use against COVID-19 might be immediate and easily accessible.

## 2. Materials and Methods

### 2.1. Plant Material

A total of 17 plant foods and derived products were selected for the current study (Figure S1). The materials were identified by the authors and acquired in a local market at Valencia (Spain) and included citrus fruit peels (sweet orange (*Citrus sinensis*), lemon (*C. limon*), lime (*C. aurantiifolia*), and grapefruit (*Citrus paradisi*)), seasoning and aromatic herbs (celery leaves and celery stalks (*Apium graveolens* var. *dulce*), parsley (*Petroselinum crispum*), dill (*Anethum graveolens*), sweet chamomile (*Marticaria chamomilla*), and dried oregano (*Origanum vulgare*)), bulbs and rhizomes (red onion (*Allium cepa*) and turmeric (*Curcuma longa*)), a succulent plant (aloe vera (*Aloe barbadensis*)), and cruciferous condiments (brown mustard seeds (*Brassica nigra*), horseradish (*Armoracia rusticana*), and commercial wasabi powder (Tokyo-Ya, S.A., Japan)). The ingredients of wasabi powder were horseradish, mustard, vitamin C, acidulant E334, wasabi aroma, and colorants E102 and E133. Dried oregano and sweet chamomile were acquired from Naturcid S.L. (Monforte del Cid, Spain), and mustard seeds were acquired from Diplan S.A. (Fuenlabrada, Spain). In addition, edible baby-leaves of wall rocket or wild rucola (*Diplotaxis erucoides* subsp. *erucoides*) were included in the study owing to their accumulation of sinigrin, which derives in allyl isothiocyanate [36,37]. Wall rocket is an herbaceous plant broadly distributed and included in the traditional Mediterranean cuisine and, for the current study, it was grown in the field as described in Guijarro-Real et al. [36]. The selection of these plant materials (Table 1) was done according to their phytochemical composition as reported in the literature, based on the recent review work by Zhou and Huang [22].

Approximately, 100 g to 200 g of material was obtained for each sample. For citrus fruits, the peels (including the flavedo and albedo) were manually separated from the flesh and used because of the higher concentrations of flavonoids in the former [38]. Celery was separated into leaves and stalks and processed separately. Turmeric and horseradish were peeled, and peels were discarded. Aloe vera leaves were separated into the outer skin and the inner parenchyma, and the former was discarded. Except for dried oregano, all materials were freeze-dried. Dried materials were ground into powder with a coffee grinder and stored at −80 °C.

### 2.2. Preparation of Methanolic Extracts

Phytochemical compounds were extracted from samples with 80% aqueous methanol, which has been proven as an appropriate solvent for the extraction of phenolic compounds [63,64] and glucosinolates [65]. For each material, four samples of 100 mg were extracted with 1 mL of 80% methanol upon sonication for 30 min, with intermediate stirring each 5 min to facilitate the extraction process. After a centrifugation step (5 min at 13,000 rpm), the plant material was re-extracted by adding 1 mL of 80% methanol. The recovered extracts of four independent replicates were combined and filtered through a 0.2 µm PTFE filter. Then, 5 mL of the combined extract for each material was dried under nitrogen and resuspended for a stock solution in 100% dimethyl sulfoxide (DMSO) to a final concentration of 50 mg mL^−1^. Extracts were kept at −80 °C until analysis.

### 2.3. FRET Assay for the Inhibition of the 3CL Protease Activity (SARS-CoV-2 3CL^Pro^)

The extracts were evaluated as inhibitors of SARS-CoV-2 3CL^Pro^ by means of an in vitro quenched fluorescence resonance energy transfer (FRET) assay using a fluorogenic substrate to measure the residual activity. For the study, the MBP-tagged 3CL Protease (SARS-CoV-2) Assay Kit (BPS Bioscience, San Diego, CA, USA) was used according to the manufacturer’s instructions. Briefly, the plant-based extracts were conveniently diluted in the 3CL^Pro^ assay buffer to an extract concentration of 2.5 mg mL^−1^. For determination of the 3CL^Pro^ activity, 5 µL of the diluted plant extracts was pre-incubated with 150 ng of the 3CL^Pro^ for 30 min. Subsequently, the fluorogenic substrate was added to a final concentration of 50 µM and the reaction was incubated for 4 h in the darkness in the presence of 1 mM 1,4-dithio-D,L-threitol (DTT). The final concentration of the plant extracts during the reaction was 500 µg mL^−1^ in a reaction volume of 25 µL. The fluorescence intensity was recorded at 470 nm after excitation at 360 nm. A positive control was included to measure the maximum activity of the protease in the absence of potential inhibitors. Moreover, an inhibition control was included by pre-incubating 150 ng of the 3CL^Pro^ with 5 µL of the inhibitor GC376 (250 µM) supplied by the manufacturer. The reaction was evaluated in four replicates for each extract.

The extracts showing significant inhibition of the 3CL^Pro^ activity (<20% of residual activity compared with the positive control) were selected for the determination of the correspondent IC_50_. The protocol followed was the same as described above, although in this case, seven concentrations of the plant-based extracts, 5.0–10–25–50–100–200–500 µg mL^−1^, were evaluated. Additionally, commercial standards of curcumin (>94% curcuminoids with >80% curcumin, CAS Number 458-37-7), sinigrin (CAS Number 3952-98-5), and allyl isothiocyanate (CAS Number 57-06-7) (Sigma-Aldrich, Saint Louis, MO, USA) were also resuspended in DMSO and tested as 3CL^Pro^ activity inhibitors at different concentrations. As for the first screening, the inhibitory capacity of the plant extracts and the commercial standards was tested using four replicates for each concentration considered.

### 2.4. Data Analysis

The fluorescence intensity measured indicated the residual activity of the protease after the co-incubation with extracts. The residual activities were provided in percentage against the maximum activity obtained in the absence of potential inhibitory compounds or extracts (100%). For each material and concentration tested, the average value and corresponding standard error were calculated (*n* = 4) using the statistics software Statgraphics Centurion XVII (Statpoint Technologies, Warrenton, VA, USA), and the significance of differences among materials was tested by one-way factorial analysis of variance. Mean separation was performed with a Student–Newman–Keuls test at a signification level of *p* < 0.05. The dose–response curves and the relative IC_50_ for extracts and standards showing significant inhibition were obtained using the GraphPad Prism 9.0.1 software (GraphPad Software, San Diego, CA, USA).

## 3. Results

### 3.1. Screening of Plant-Based Extracts for Inhibition of SARS-CoV-2 3CL^Pro^ Activity

The inhibition of SARS-CoV-2 3CL^Pro^ activity by the 17 extracts was tested at a final concentration of 500 µg mL^−1^. The extracts of lime peel and chamomile produced signal interferences and were discarded from the analysis. It was noted, however, that the co-incubation of the 3CL^Pro^ with the chamomile extracts produced a reduction of the protease activity even if a signal interference was observed (Figure S2).

**Figure 1:**
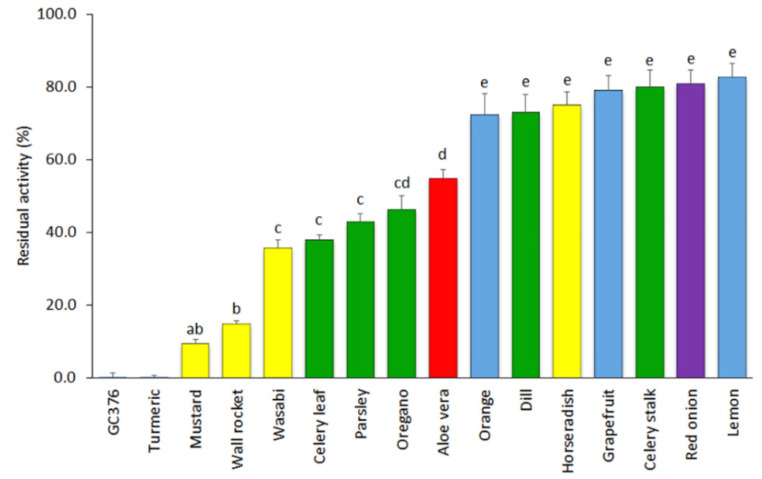
Mean values and standard errors (SEs) for the residual activity of SARS-CoV-2 3CL^Pro^ in the presence of the fifteen plant-based extracts at a concentration of 500 µg mL^−1^ and the inhibitor control GC376 at a concentration of 50 µM (*n* = 4). Extracts included were obtained from citrus fruit peels (blue), seasoning and aromatic herbs (green), bulbs and rhizomes (purple; includes turmeric), a succulent plant (red), and cruciferous herbs and condiments (yellow). Both the turmeric extract and the inhibitor GC376 produced a complete inhibition of the 3CL^Pro^. Different letters indicate significant differences in the 3CL^Pro^ residual activity according to a Student–Newman–Keuls test at *p* < 0.05.

The results of the inhibitory activity of SARS-CoV-2 3CL^Pro^ of the remaining 15 extracts tested at a concentration of 500 µg mL^−1^ are displayed in Figure 1. Seven of the materials showed low inhibitory capacity and led to average residual protease activities over 70%. This group included extracts of grapefruit, lemon, and orange fruit peels (72.4–82.8% of mean residual activities); red onion (80.9%); celery stalk (79.9%); horseradish (75.0%); and dill (73.1%). Five other materials displayed intermediate inhibitory capacity (35–55%), including extracts of celery leaves (38.0%), parsley (42.8%), and oregano (46.3%) herbs; aloe vera leaves (54.8%); and the wasabi powder (35.8%). Finally, three extracts were found to show high inhibitory capacity and included mustard seeds (9.4%), wall rocket (14.9%), and turmeric, the latter showing a complete inhibitory capacity (0.0% of 3CL^Pro^ residual activity) at the concentration of 500 µg mL^−1^.

### 3.2. Determination of the IC_50_ Values of Extracts with High SARS-CoV-2 3CL^Pro^ Activity Inhibition

According to the initial screening at 500 µg mL^−1^, the mustard seeds, wall rocket leaves, and turmeric powder extracts were selected owing to their high SARS-CoV-2 3CL^Pro^ activity inhibitory potential. For these materials, the dose-dependent inhibitory curves were obtained and the corresponding IC_50_ values were calculated by testing the plant extracts at eight final concentrations ranging between 5.0 and 500 µg mL^−1^.

**Figure 2:**
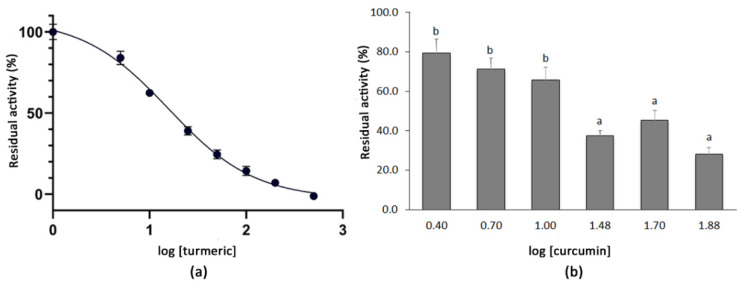
(**a**) Dose–response curve for the methanolic extract of turmeric powder. Each point represents the average value and SE for the residual activity of the 3CL^Pro^ (%) at extract concentrations between 5.0 and 500 µg mL^−1^ (*n* = 4). The 100% of residual activity considers the activity of the 3CL^Pro^ when no inhibitor is added. (**b**) Mean residual activity and SE of 3CL^Pro^ (%) determined after the co-incubation with five different concentrations of commercial curcumin (2.5–75 µg mL^−1^) (*n* = 4). Different letters indicate significant differences in the 3CL^Pro^ residual activity according to a Student–Newman–Keuls test at *p* < 0.05.

The dose-dependent inhibition curve for the turmeric powder was obtained by testing the residual activity of the SARS-CoV-2 3CL^Pro^ after the co-incubation with the plant extracts at the final concentrations of 5.0–10–25–50–100–200–500 µg mL^−1^. The logarithmic dose–response curve was fitted to a sigmoidal curve with an *r^2^* value of 0.976 and an IC_50_ for turmeric of 15.74 µg mL^−1^ was determined. The highest concentrations tested, i.e., from 100 µg mL^−1^ (log [turmeric] = 2.0) to 500 µg mL^−1^ (log [turmeric] = 2.7), resulted in a very high inhibition of the SARS-CoV-2 3CL^Pro^ activity (Figure 2). In order to test whether the curcumin present in the turmeric material accounted for such inhibition, an additional analysis using commercial curcumin (>94% curcuminoids, >80% curcumin) was conducted at concentrations of 2.5–75 µg mL^−1^ (log [curcumin] = 0.4–1.9). Higher concentrations of the compound could not be tested because of its insolubility in the reaction buffer. As expected, the co-incubation of 3CL^Pro^ with curcumin also showed a positive effect on the protease inhibition. Thus, concentrations over 30 µg mL^−1^ (log [curcumin] = 1.5) produced a residual protease activity below 50%, which decreased to 28.1% when the compound was tested at 75 µg mL^−1^ (log [curcumin] = 1.9) (Figure 2).

**Figure 3:**
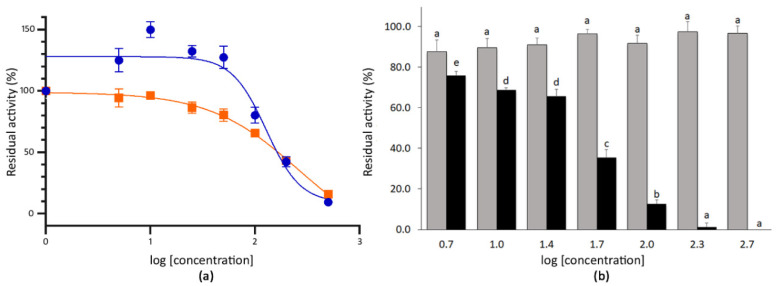
(**a**) Dose–response curve for the methanolic extracts of mustard seeds (blue circles) and wall rocket leaves (orange squares). Each point represents the average value and SE for the residual activity of the 3CL^Pro^ (%) at extract concentrations between 5.0 and 500 µg mL^−1^ (*n* = 4). The 100% of residual activity is the activity of the 3CL^Pro^ when no inhibitor is added. (**b**) Mean residual activity and SE of 3CL^Pro^ (%) determined after the co-incubation with 5.0–500 µg mL^−1^ of sinigrin (grey) and allyl isothiocyanate (black) standards (*n* = 4). Different letters indicate significant differences in the 3CLPro residual activity for sinigrin or allyl isothiocyanate treatments according to two separate Student–Newman–Keuls tests at *p* < 0.05.

The extracts of mustard seeds showed strong inhibitory capacity at the highest doses tested. Thus, the co-incubation with 200 µg mL^−1^ (log (mustard) = 2.3) and 500 µg mL^−1^ (log (mustard) = 2.7) of the extract reduced the activity of SARS-CoV-2 3CL^Pro^ to 42.1% and 9.4%, respectively (Figure 3). By contrast, the co-incubation with 100 µg mL^−1^ (log (mustard) = 2.0) of mustard seeds extract only inhibited such activity by 19.9% (residual activity = 80.1%). The IC_50_ for the mustard seeds extract was 128.1 µg mL^−1^ (*r^2^* value 0.865) (Figure 3). In the case of wall rocket extracts, the co-incubation produced residual activities of the 3CL^Pro^ ranging between 94.3% and 15.8% for 5.0 µg mL^−1^ (log (wall rocket) = 0.7) and 500 µg mL^−1^ (log (wall rocket) = 2.7) of extract, respectively. The co-incubation with 200 µg mL^−1^ of extract produced an inhibitory response of 56.8% compared with the absence of extract. For this material, the IC_50_ estimated from the dose–response curve was 257.4 µg mL^−1^ (*r^2^* value 0.937) (Figure 3).

Given that both mustard and wall rocket were selected for containing glucosinolates, particularly sinigrin, both sinigrin and its hydrolytic derivative were evaluated for their SARS-CoV-2 3CL^Pro^ inhibition activity. Allyl isothiocyanate is naturally released in plants from its precursor owing to the activity of the endogenous plant myrosinases [66]. The co-incubation of the 3CL^Pro^ with the purified sinigrin did not result in a significant reduction of the protease activity even at the highest concentration tested, 500 µg mL^−1^. By contrast, the co-incubation with allyl isothiocyanate at 500 µg mL^−1^ (log (allyl isothiocyanate) = 2.7) resulted in a complete inhibition of the 3CL^Pro^ activity. Therefore, the dose–response curve was obtained for allyl isothiocyanate. The response fitted to a sigmoidal curve (*r^2^* value 0.943) with an estimated IC_50_ of 41.43 µg mL^−1^ allyl isothiocyanate standard (Figure 3).

## 4. Discussion

The recent COVID-19 disease pandemic caused by the SARS-CoV-2 coronavirus has resulted in a global health crisis. Although other coronaviruses were responsible for previous outbreaks in the last two decades [67], the fast spread of SARS-CoV-2 has demonstrated the threat to human health of new coronaviruses. One year after the disease was declared as a pandemic [68], COVID-19 is still causing thousands of daily deaths and no effective treatments apart from preventive vaccination are reported for fighting the infection process. Obtaining effective treatments against SARS-CoV-2 is urgently needed because, in combination with vaccines, they may become a first line therapy not only for current SARS-CoV-2 strains, but also for new ones.

The 3CL^Pro^ is considered as one of the most promising targets for the development of antiviral drugs against coronaviruses [69]. The three-dimensional structure of SARS-CoV-2 3CL^Pro^ was unraveled by Zhang et al. [12]. The enzyme has an identity percentage of 96% with SARS-CoV 3CL^Pro^, thus it has been inferred that both proteases have very similar specificities [70,71]. These works suggest that molecules and extracts that displayed inhibitory activity against SARS-CoV 3CL^Pro^ might be also of interest to inhibit the activity of SARS-CoV-2 3CL^Pro^. In this context, our work used a targeted approach based on previous information on inhibitors SARS-CoV 3CL^Pro^ [22]. For the current study, plant-based extracts potentially promising for the inhibition of SARS-CoV-2 3CL^Pro^ activity were tested. Previous studies with plant-based extracts have been conducted in the past to test their inhibitory capacity against SARS-CoV 3CL^Pro^, with promising results [15,16,72].

The first screening with plant extracts at 500 µg mL^−1^ revealed that the extracts of the three citrus fruits peels produced a low reduction of the protease activity. Flavonoids present in citrus fruits have been suggested as potential antivirals against coronaviruses and in therapeutics fighting the inflammation process that can derive in more severe symptoms of the viral disease [73,74]. Recent works based on computational molecular docking pointed to the flavanone hesperidin, which is the glycosylated form of hesperetin and the main form in which this flavonoid is found in citrus fruits, as one natural compound displaying high capacity for binding to the SARS-CoV-2 3CL^Pro^ [71,75]. However, our results showed that citrus fruit peels, which contain high concentrations of hesperidin [44], had a low in vitro inhibitory activity against SARS-CoV-2 3CL^Pro^.

Interestingly, extracts of turmeric powder, mustard seeds, and wall rocket leaves produced the highest reduction in SARS-CoV-2 3CL^Pro^ activity. Turmeric rhizomes are rich in the flavonoid curcumin and its derivatives, natural compounds that have been also considered in molecular docking studies for their potential inhibitory capacity against SARS-CoV-2 3CL^Pro^ [75,76,77]. According to these studies, curcumin and its derivatives can be considered as natural compounds with potential as 3CL^Pro^ inhibitors, although the same works showed that other flavonoids might have higher potential inhibitory activity according to the docking results. In concordance, our FRET assay revealed a high inhibitory capacity of the turmeric rhizomes extracts against the 3CL^Pro^ activity. The turmeric extracts were, in fact, the most effective among all the plant-based extracts evaluated in the inhibitory activity. In this respect, other plant-based extracts were evaluated in the past against SARS-CoV 3CL^Pro^ activity and showed lower inhibitory capacity. Ryu et al. [72] found that ethanolic extracts of *Torreya nucifera* tested at 100 µg mL^−1^ resulted in 62% of activity inhibition against SARS-CoV 3CL^Pro^, while in our case, the same concentration of turmeric extract resulted in a reduction of 86% for SARS-CoV-2 3CL^Pro^ activity. In another study, an IC_50_ was determined for SARS-CoV 3CL^Pro^ activity that ranged between 25 and 70 µg mL^−1^ for black and Puer tea extracts [15], while for the turmeric extracts, we found an IC_50_ of 1.6- to 4.5-fold lower than these against SARS-CoV-2 3CL^Pro^.

The concentration of curcumin in turmeric powder is generally around 3% or lower [78], while the concentration of other curcuminoids is even lower [79]. However, commercial curcumin (>80% curcumin) even at concentrations of 75 µg mL^−1^, which is several-fold the expected concentration of curcumin in the turmeric extract at 500 µg mL^−1^, did not cause a complete inhibition of SARS-CoV-2 3CL^Pro^ activity. This result suggests that other components of turmeric or their synergetic interaction may have a major contribution to the inhibition of SARS-CoV-2 3CL^Pro^ activity. In this sense, turmeric rhizomes also contain the curcuminoid demethoxycurcumin [56], which has also been described as a potential inhibitor against SARS-CoV-2 3CL^Pro^, even showing greater affinity than curcumin for SARS-CoV-2 3CL^Pro^ in the docking analysis of Khaerunnisa et al. [77]. Furthermore, turmeric powder also contains sesquiterpenoids [79] and quercetin derivatives [80], the latter also being considered as a potential inhibitor of SARS-CoV-2 3CL^Pro^ [77,81]. Thus, the inhibitor capacity of turmeric extracts might be the result of a synergistic activity of several compounds. Overall, our results indicate that extracts of turmeric are a strong candidate for being tested for inhibiting the in vivo replication of SARS-CoV-2. Moreover, considering these results, further studies should be addressed to characterize the methanolic extracts of turmeric powder and to individually test the compounds present.

On the other hand, two cruciferous extracts (from mustard seeds and wall rocket) that are rich in sinigrin [36,57,58,61] were found to exert a strong inhibition of SARS-CoV-2 3CL^Pro^ activity. Sinigrin is the precursor glucosinolate of allyl isothiocyanate, a reactive molecule displaying antimicrobial [82,83] and anticarcinogenic properties [84,85,86]. By contrast, references to the potential of either sinigrin or allyl isothiocyanate as antivirals are more limited [16]. These latter authors found that sinigrin had good potential as an inhibitor against SARS-CoV 3CL^Pro^. In our case, we found that sinigrin has a low effect as in vitro inhibitor of SARS-CoV-2 3CL^Pro^, while its derivative allyl isothiocyanate is a powerful inhibitor of SARS-CoV-2 3CL^Pro^ activity. In this way, isothiocyanate metabolites might be responsible, at least in part, for the antiviral properties of these cruciferous plants extracts, which is probably related to their chemical structure, i.e., organosulfur molecules acting as H2S donors [87]. In fact, Blanchard et al. [14] previously reported a good response of sulfur-containing compounds against SARS-CoV 3CL^Pro^ activity owing to their capacity of forming covalent adducts between their electrophilic group and a nucleophile side chain of the protease. Therefore, our results suggest that the sinigrin derivative allyl isothiocyanate is a good candidate molecule for being tested in vivo for inhibiting SARS-CoV-2 3CL^Pro^ activity. However, the high cytotoxicity of allyl isothiocyanate may preclude its practical use [83], which would depend on the concentration required. In this sense, further analysis of the methanolic extracts for mustard and wall rocket would increase the knowledge regarding the concentration at which these compounds are found, and the possible presence of other metabolites also exerting potential inhibitory activities.

This work is a first targeted approach for the evaluation of plant extracts and containing natural compounds for the development of prophylaxis, adjuvant therapies, and drug treatments aimed at inhibiting the activity of SARS-CoV-2 3CL^Pro^. Extracts of turmeric have been identified as a candidate plant extract for reducing SARS-CoV-2 3CL^Pro^ activity, which could eventually affect the viral replication. In addition, other plant extracts, such as those of cruciferous plants containing sinigrin, which, after consumption, is degraded to allyl isothiocyanate, might be considered as well for their inhibition of SARS-CoV-2 3CL^Pro^ activity. The current study provides information that can help in the search for treatments against COVID-19, acting as a basis for future chemical, in vivo, and clinical trials. Thus, further studies following the results of this work should be addressed: (1) to chemically characterize the plant extracts with high potential inhibitory activity and to re-evaluate this capacity after the fractionation of the extracts, thus allowing the identification of the biomolecules responsible of such activity and possible synergistic effects; and (2) to evaluate this potential inhibition in cell-based studies where the virus, host cell, and plant extract interact, also determining the toxicity limit prior to conducting other pre-clinical and clinical trials.

## Figures and Tables

**Table 1:** Selected phytochemical compounds reported as potential natural inhibitors of SARS-CoV-2 3CL^Pro^ activity and naturally present in the plant materials used in the current study.

Compound	Chemical Family	Plant Material	References
Hesperetin	Flavonoids	Grapefruit Lemon Lime Orange	[39,40,41,42] [43,44] [45,46] [39,40]
Luteolin	Flavonoids	Celery Oregano	[47,48,49] [50,51]
Quercetin	Flavonoids	Dill Red onion	[52] [48,49]
Apigenin	Flavonoids	Celery Chamomile Oregano Parsley	[47,48] [53,54] [50,51] [47,52]
Curcumin	Curcuminoids	Turmeric	[55,56]
Sinigrin	Glucosinolates	Mustard Horseradish Wasabi condiment ^1^ Wall rocket	[57,58] [59,60] [37] [36,61]
Aloe emodin	Anthraquinones	Aloe vera	[62]

^1^ The wasabi was obtained as commercial wasabi powder; for ingredients, refer to Section 2.1.

## Data Availability

Data are contained within the article or Supplementary Materials.

## Supplementary Materials

The following are available online at https://www.mdpi.com/article/10.3390/foods10071503/s1, Figure S1: Materials used for the analysis, from left to right and top to bottom: orange peel, lemon peel, lime peel, grapefruit peel, celery leaves, celery stalk, parsley leaves, dill leaves, dried oregano leaves, red onion bulb, turmeric rhizomes, aloe leaf, chamomile, mustard seeds, horseradish root, wasabi powder, and wall rocket leaves; Figure S2: Signal recorded at wavelengths between 400 and 550 nm for the residual activity of the 3CL^Pro^ without inhibition (Control), after co-incubation with chamomile extracts at concentrations of 200 µg mL^−1^ or 500 µg mL^−1^, after co-incubation with the inhibitor GC376 at 50 µM (GC376), and the signal of the raw chamomile extract at a concentration of 500 µg mL^−1^ without incubation with 3CL^Pro^ (Raw).
