# Supplementary figures and images for: Potential In Vitro Inhibition of Selected Plant Extracts against SARS-CoV-2 Chymotripsin-Like Protease (3CLPro) Activity
Source: Foods. 2021 Jun 29;10(7):1503. doi: 10.3390/foods10071503 (PMC8304378; doi:10.3390/foods10071503)

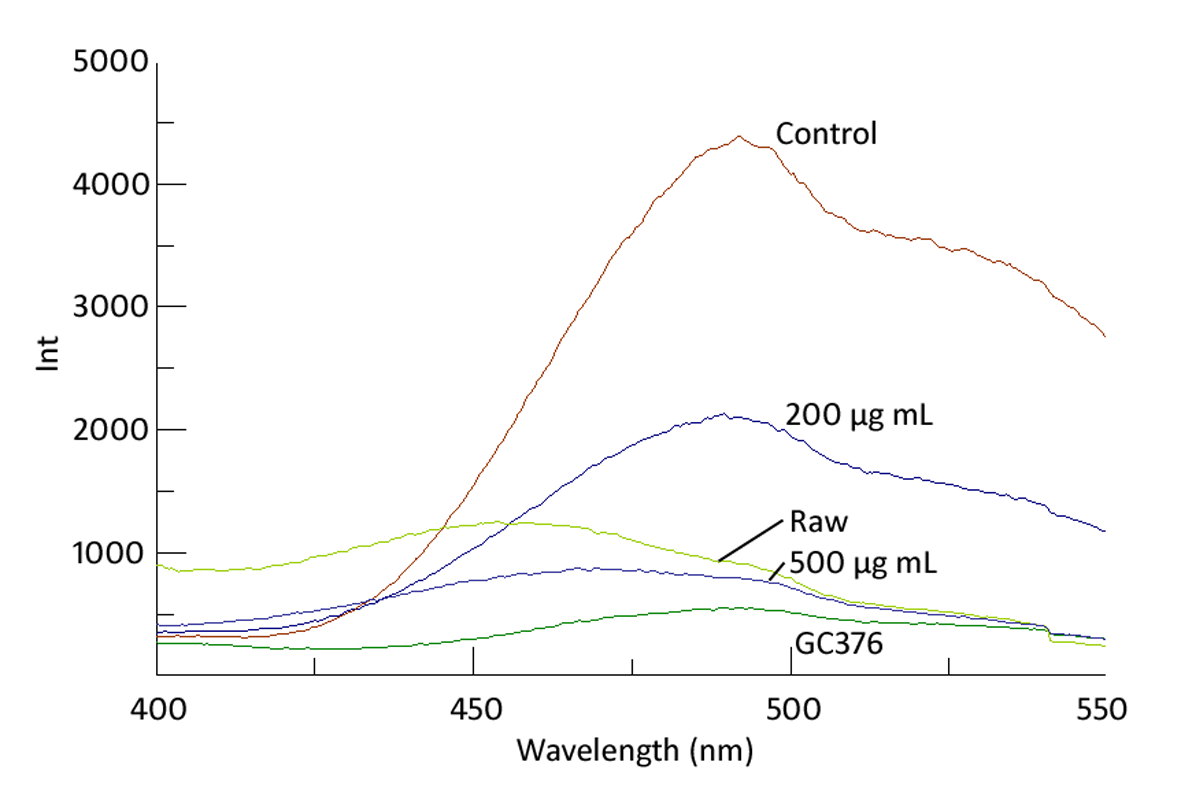

Supplement: Supplementary file 1 [file foods-10-01503-s001.zip › FigureS2.tif]

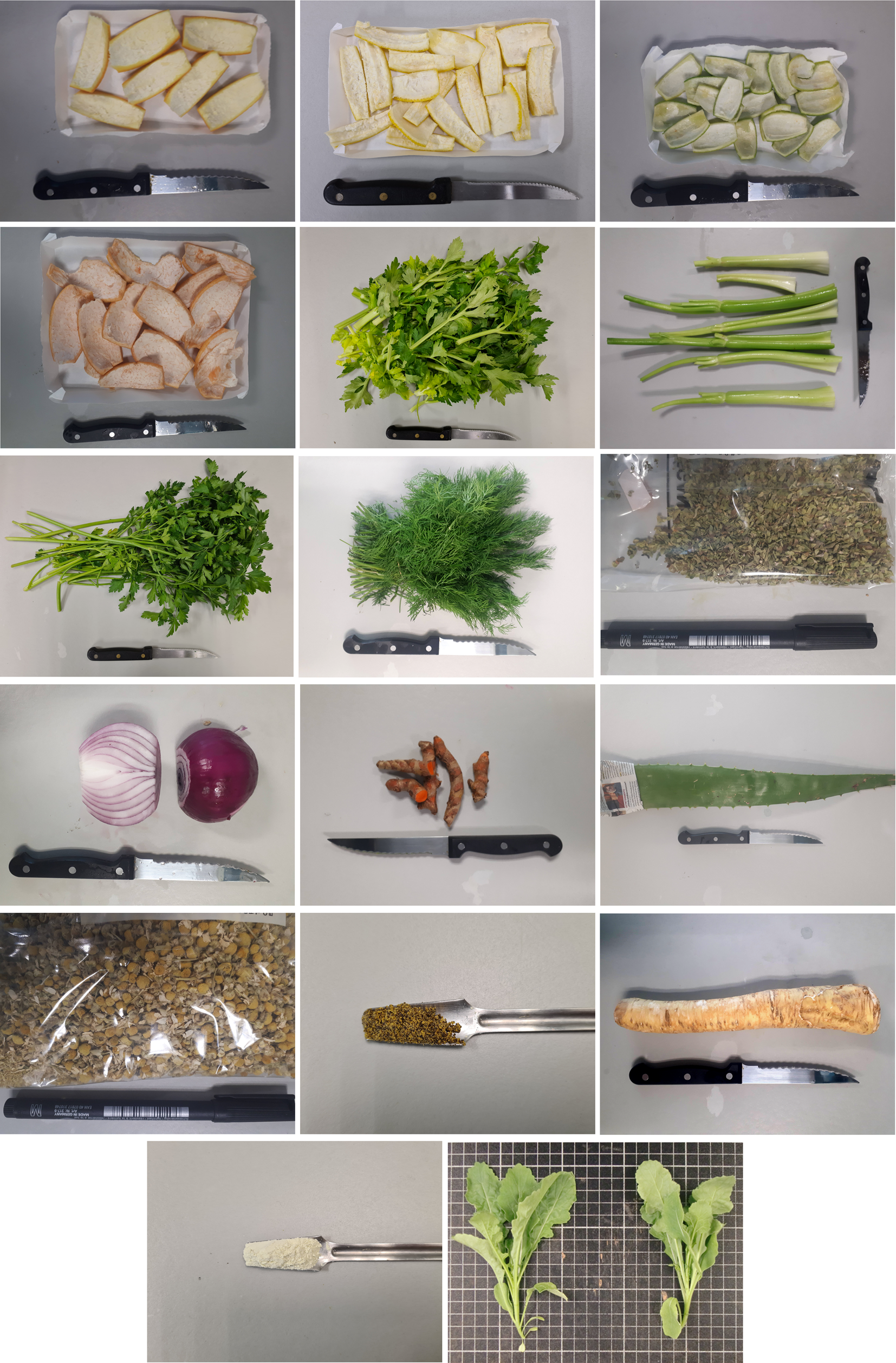

Supplement: Supplementary file 1 [file foods-10-01503-s001.zip › FigureS1.tif]
